# Draft Genome Sequence of Clostridium butyricum Strain 16-3, Isolated from Neonatal Feces

**DOI:** 10.1128/mra.00163-22

**Published:** 2022-07-27

**Authors:** Jang-In Shin, Man-Seok Bang, Gi-Su Lee, Ha-Na Kim, Chung-Hun Oh

**Affiliations:** a Department of Oral Physiology, College of Dentistry, Dankook University, Cheonan, Choongnam, Republic of Korea; b Department of Medical Laser, Graduate School, Dankook University, Cheonan, Choongnam, Republic of Korea; University of Maryland School of Medicine

## Abstract

Here, we report the genome sequence of Clostridium butyricum strain 16-3, which was isolated from infant feces. The genome contains circular contigs of 3,861,515 bp and 769,300 bp, with G+C contents of 28.8% and 28.3%, respectively.

## ANNOUNCEMENT

Clostridium butyricum roles range from a promising biofuel producer (1) to a neurotoxigenic pathogen (2). C. butyricum is a butyric acid-producing, Gram-positive anaerobe that is found in soil and in the intestines of healthy animals and humans (3); it has been shown to have beneficial effects on animal and human health and performance, and it is commonly used as a feed additive in Asia and Europe (4–6). Here, we present the genome sequence of Clostridium butyricum strain 16-3, which was isolated from infant feces.

Fecal samples were collected from breastfeeding infants less than 3 months of age at a hospital and postpartum care center as described previously (7). For the isolation of C. butyricum, collected fecal samples were incubated at 30°C in an anaerobic chamber (Forma anaerobic system; Thermo Fisher Scientific Inc., Waltham, MA, USA) for 3 days. The samples were serially diluted and plated on reinforced clostridial medium (RCM) (BD) in an anaerobic chamber (Forma anaerobic system) using a gas phase of N_2_/H_2_/CO_2_ (80:10:10 [vol/vol/vol]) at 30°C for 48 h. The colonies were purified on the agar medium. Isolates showing a bubble with soap skin were selected, and their biochemical profiles were confirmed using an API 20A kit (bioMérieux, UK). C. butyricum strain16-3, which showed the active formation of gas bubbles, was chosen, and its identification was confirmed by whole-genome sequencing.

The total genomic DNA of C. butyricum strain16-3 was extracted from RCM broth cultures that had been grown at 25°C for 2 days using the Wizard genomic DNA purification kit (Promega, USA) according to the protocol recommended by the manufacturer. The quantity and quality of the isolated DNA were determined using a NanoDrop spectrophotometer (Thermo Fisher Scientific). To sequence the whole genome of Clostridium butyricum strain 16-3, a SMRTbell library with a 15- to 20-kb insert size (BluePippin size selection system) was constructed with Pacific Biosciences (PacBio) DNA template preparation kit v1.0. The genome was sequenced with the RS II sequencing platform (PacBio, USA) using single-molecule real-time (SMRT) cell 8Pac v3 and DNA polymerase binding kit P6 reagents by Macrogen (Seoul, Republic of Korea) (8).

In total, 202,356 PacBio subreads (average subread length, 4,223 bp; *N*_50_, 6,195 bp) were generated. PacBio reads were assembled using the RS Hierarchical Genome Assembly Process (HGAP) protocol v3.0 (9–11). Default parameters were used except where otherwise noted. When the contig ends overlapped, contigs were connected to form a circular DNA.

The result of the assembly was two circular contigs, consisting of 3,861,515 bp (G+C content, 28.8%; coverage, 124×) (GenBank accession number CP053292.1) and 769,300 bp (G+C content, 28.3%; coverage, 116×) (GenBank accession number CP053293.1). Genome annotation using Prokka v1.13 (8) and the NCBI Prokaryotic Genome Annotation Pipeline (PGAP) predicted a total of 4,223 genes, including 4,020 protein coding sequences (CDSs), 87 tRNAs, 33 rRNAs, 4 noncoding RNAs, and 79 pseudogenes.

### Data availability.

The complete genome sequence of C. butyricum 16-3 was deposited under GenBank accession numbers CP053292.1 and CP053293.1. The associated BioProject, BioSample, and SRA accession numbers are PRJNA630842, SAMN14846469, and SRX8334862, respectively.
